# Clinical and Neurochemical Effects of Transcranial Magnetic Stimulation (TMS) in Multiple Sclerosis: A Study Protocol for a Randomized Clinical Trial

**DOI:** 10.3389/fneur.2020.00750

**Published:** 2020-08-11

**Authors:** Eduardo Agüera, Javier Caballero-Villarraso, Montserrat Feijóo, Begoña M. Escribano, Cristina Conde, María C. Bahamonde, Ana I. Giraldo, Elier Paz-Rojas, Isaac Túnez

**Affiliations:** ^1^Instituto Maimónides de Investigación Biomédica de Córdoba (IMIBIC), Córdoba, Spain; ^2^Unidad de Gestión Clínica de Neurología, Hospital Universitario Reina Sofía, Córdoba, Spain; ^3^Departmento de Bioquímica y Biología Molecular, Facultad de Medicina y Enfermería, Universidad de Córdoba, Córdoba, Spain; ^4^Unidad de Gestión Clínica de Análisis Clínicos, Hospital Universitario Reina Sofía, Córdoba, Spain; ^5^Departamento de Biología Celular, Fisiología e Inmunología, Universidad de Córdoba, Córdoba, Spain; ^6^Canvax Biotech S.L., Córdoba, Spain

**Keywords:** clinical trial, multiple sclerosis, neurodegenerative diseases, transcranial magnetic stimulation, neuroplasticity, neurochemistry

## Abstract

**Background:** Transcranial Magnetic Stimulation (TMS) is a technique based on the principles of electromagnetic induction. It applies pulses of magnetic radiation that penetrate the brain tissue, and it is a non-invasive, painless, and practically innocuous procedure. Previous studies advocate the therapeutic capacity of TMS in several neurodegenerative and psychiatric processes, both in animal models and in human studies. Its uses in Parkinson's disease, Alzheimer's disease and in Huntington's chorea have shown improvement in the symptomatology and in the molecular profile, and even in the cellular density of the brain. Consequently, the extrapolation of these TMS results in the aforementioned neurodegenerative disease to other entities with etiopathogenic and clinical analogy would raise the relevance and feasibility of its use in multiple sclerosis (MS). The overall objective will be to demonstrate the effectiveness of the TMS in terms of safety and clinical improvement, as well as to observe the molecular changes in relation to the treatment.

**Methods and Design:** Phase II clinical trial, unicentric, controlled, randomized, single blind. A total of 90 patients diagnosed with relapsing-remitting multiple sclerosis (RRMS) who meet all the inclusion criteria and do not present any of the exclusion criteria that are established and from which clinically evaluable results can be obtained. The patients included will be assigned under the 1:1:1 randomization formula, constituting three groups for the present study: 30 patients treated with natalizumab + white (placebo) + 30 patients treated with natalizumab + TMS (1 Hz) + 30 patients treated with natalizumab + TMS (5 Hz).

**Discussion:** Results of this study will inform on the efficiency of the TMS for the treatment of MS. The expected results are that TMS is a useful therapeutic resource to improve clinical status (main parameters) and neurochemical profile (surrogate parameters); both types of parameters will be checked.

**Ethics and Dissemination:** The study is approved by the Local Ethics Committee and registered in https://clinicaltrials.gov (NCT04062331). Dissemination will include submission to a peer-reviewed journal, patients, associations of sick people and family members, healthcare magazines and congress presentations.

**Trial Registration:** ClinicalTrials.gov ID: NCT04062331 (registration date: 19^th^/ August/2019).

**Version Identifier:** EMTr-EMRR, ver-3, 21/11/2017.

## Introduction

Transcranial magnetic stimulation (TMS) is based on the principles of Maxwell's electromagnetism, by which an electric field is capable of generating a magnetic field perpendicular to it and vice versa. This peculiar therapeutic strategy with more than two decades of application was authorized in 2008 for the treatment of refractory major depression by the Food and Drug Administration (FDA) of the United States and later by the European Medicines Agency (EMEA). At present, its approval for diseases such as schizophrenia (1), neuropathic pain (2), effects of ischemic stroke (3) and amyotrophic lateral sclerosis (ALS) (4) and multiple sclerosis (MS) (5, 6), among others, is under study.

In recent years, the evidences have revealed the therapeutic potential of TMS in the treatment of Alzheimer's disease, improving psychomotor skills and the memory of affected patients (7–9). Similarly, it has been observed that the application of TMS in Parkinson's patients produces a symptomatic improvement at different levels, such as the decrease of resting tremor, spastic rigidity and bradykinesia (10–15). Furthermore, some studies have shown a certain degree of improvement when using TMS to reduce neuropathic pain (16, 17) or the severity of spasticity subsequent to an ischemic stroke (18–22).

Experimental models confirm the usefulness of this therapeutic resource at different levels, showing even what could be the intimate mechanisms (at cellular and molecular level) involved in the therapeutic effect that the induction of the electromagnetic current that TMS produces. As an example of this we can find studies in murine models of Parkinson's disease (induced by 6-hydroxydopamine) and Alzheimer's disease that show a behavioral improvement after the administration of TMS. Such clinical improvement would be related to a decrease in circulating levels of cyclooxygenase-2 and TNF-alpha, as well as an increase in subventricular neurogenesis (9, 23–26).

In this regard, data published by our group show how the application of TMS on an animal model of Huntington's chorea (induced by 3-nitropropionic acid) (27), show not only an improvement in the clinical aspects, but also a quantitative cellular increase, corroborated by neurohistological studies, as well as a decrease in oxidative and cellular damage (28–31). Our group has also recently participated in studies that relate the administration of TMS to an improvement in parameters that characteristically deteriorate with age, such as sleep quality, learning ability and memory; such findings have been observed in an experimental model of an elderly subject developed in rodents of the aged Octodon degus type (32, 33). Finally, in line with the above, we could add the results obtained by our group in an experimental model of Wistar rat major depression (induced by olfactory bulbectomy), in which after treatment with TMS there is an increase in the levels of serotonin and in the cellularity of the brain, which at a symptomatic level correlates with an improvement in the depression and anxiety scales of the animal (34, 35).

The aforementioned entities have analogies at the symptomatological and semiological level with other neurodegenerative diseases, such as multiple sclerosis (MS). In some of these entities, it could even be said that they also present some factors common to MS at the etiological or pathogenic level, with molecular or cellular processes of some similarity being involved. Multiple sclerosis (MS) is a demyelinating disease of neuroinflammatory type and autoimmune nature. It is the main cause of non-traumatic invalidating neurological disease in young adults, constituting the first cause of disability acquired between 20 and 40 years of age in Spain. It leads to a significant reduction in the quality of life, a high health cost and a reduction in the productive working life of patients (due to possible absenteeism and reduction of the patient's active professional life). It presents a higher frequency in women than in men (3:2) and according to recent data, it is estimated that in recent years its prevalence has increased to 120 cases per 100,000 inhabitants. It denotes a geographical distribution, concentrating more casuistry in western countries, increasing its frequency as its latitude moves away from the equator. Its clinical manifestations can be very varied, both in its debut and in possible subsequent outbreaks, often manifesting itself with asthenia, muscle weakness, ataxia, spasticity, dysarthria and dysphagia, among other symptoms and signs. Its etiology is still unknown, although several studies have linked the origin of MS with certain infections, vitamin D deficiency, dietary factors and genetic susceptibility, among other possible factors. Similarly, in its pathogenesis the presence of inflammatory processes and oxidative stress has been identified, among other elements. In this regard, recent studies of our group have found an important role of oxidative damage in the origin and evolution of this entity, as well as of inadequate (decreased) levels of melatonin; This is an endogenous neurohormone regulator of sleep (which is usually altered in these patients) and of high antioxidant power. Due to the heterogeneity of forms and manifestations of MS, the treatment must be individualized. However, it should be noted that currently there is no curative treatment for this condition. Corticosteroids are used to treat acute attacks, while maintenance treatment is based mainly on the use of immunomodulators, among which interferons and glatiramer acetate are prevalent. Fifteen years ago, the FDA authorized the use of Natalizumab (a monoclonal antibody), and Alemtuzumab 5 years ago, mainly for forms that occur in the form of recurrent outbreaks that do not respond to other treatments. Other immunomodulatory strategies even try to stop the evolution by promoting remyelination processes; such as in the case of anti-LINGO, which is currently being used in phase II-III clinical trials (36).

Consequently, the absence of a definitive or curative treatment for this disease encourages us to seek the identification and validation of alternative therapeutic targets, as well as the evaluation of new treatment options for MS patients. From this perspective, the use of TMS in patients with MS is considered. TMS has proven to be an effective therapeutic resource in certain neurodegenerative and neuropsychiatric pathologies without defined therapeutic options or in such situations in which with available treatments the disease is refractory to treatment, as it can occur in cases of major depression. Along with these considerations, TMS has proven to be a relatively innocuous procedure in terms of adverse effects. To this we should add that it is a non-invasive technique that is easy to apply as well as being safe. The suggested side effects are extremely infrequent, given that only certain cases of headache and (even more rarely) some seizure episodes, are described (37).

In the decade of the 90s, Sandyk showed in different studies how electromagnetic stimulation manages to improve the visual and cognitive deficit associated with MS, as well as reducing the symptomatic exacerbation (of the general case) associated with the premenstrual state (38). Professor Centonze's group, since the beginning of the XXI century has been studying the effect of the TMS on the spasticity typical of the patients affected by MS. This has shown how the application of TMS at 5 Hz in the areas of the motor cortex for 2 weeks, significantly improved spasticity of the lower limbs. This effect was maintained until 7 days after the last session (39). Additionally, they described the relationship of this pattern with improvement in sphincter control, cerebellar symptoms, and manual dexterity (40). In a 2010 article, the same group showed how another pattern; in this case intermittent theta burst stimulation (iTBS), also produced lower limb spasticity attenuation when applying TMS in patients with MS, while describing its ease of application, safety and tolerance (41). Elzamarany et al. presented the results of the application of TMS in the sagittal mid axis of the motor cortex at 5 Hz and 900 pulses of application in patients with relapsing-remitting MS (RRMS) and secondarily progressive MS (PMSS). After that the patients showed a clear improvement, more evident in the forms of RRMS (42).

Data derived from the experimental model of demyelination of the corpus callosum by inoculation of lysophosphatidylcholine, show how the application of electromagnetism (with the same experimental protocol as that used by our group in an experimental model of Huntington's disease), induces improvement in the symptoms of the animal, which is associated with neurogenesis and a reduction in inflammatory phenomena. In this line, published data from our group show how the application of TMS in the model similar to RRMS in rats with experimental autoimmune encephalomyelitis (EAE) show a symptomatic improvement in the mobility scale, as well as in oxidative and cellular damage, decreasing the degree of cerebral astrocytosis (43–46).

However, despite the vast information available in the scientific literature about the potential therapeutic use of TMS, pointing out its effectiveness and safety, the following four premises must be borne in mind (27, 34): (i) The mechanisms of action that achieve the therapeutic effects described are not well-known at the molecular level. (ii) There is a wide variety of administration protocols, which reveals absence of consensus and standardization of guidelines. (iii) It is not known exactly how long the effects of TMS will last after the administration of a cycle of sessions. (iv) Any possible change in any of its variables (frequency, intensity, number of pulses, etc.) could imply the creation of a new protocol and, therefore, a new therapeutic strategy in itself.

All these arguments reveal the relevance of conducting clinical trials that result in understanding the effectiveness of TMS in the treatment of MS. In this way, the presumed potentialities mentioned above could be verified, as well as the elaboration of different specific protocols whenever possible formulas of optimisation of administration guidelines are known (in terms of intensity, number, and chronology of sessions). For these purposes, the present study proposes the comparative application of TMS of low frequency (1 Hz) and high frequency (5 Hz) in patients affected by relapsing-remitting MS, compared to a treatment in use. It is based on the hypothesis that the administration of TMS in MS aim to achieve neuromodulation for a therapeutic purpose in patients with relapsing-remitting multiple sclerosis (RRMS), and this implies a neuroprotective effect against the progression of the disease, resulting in a clinical improvement (attenuation of symptoms and signs, as direct measures of the therapeutic effect) and a biochemistry improvement (decrease of serum oxidative stress molecules and acute phase reactants, as indirect measures).

## Methods and Analysis

### Study Design

**Figure 1:**
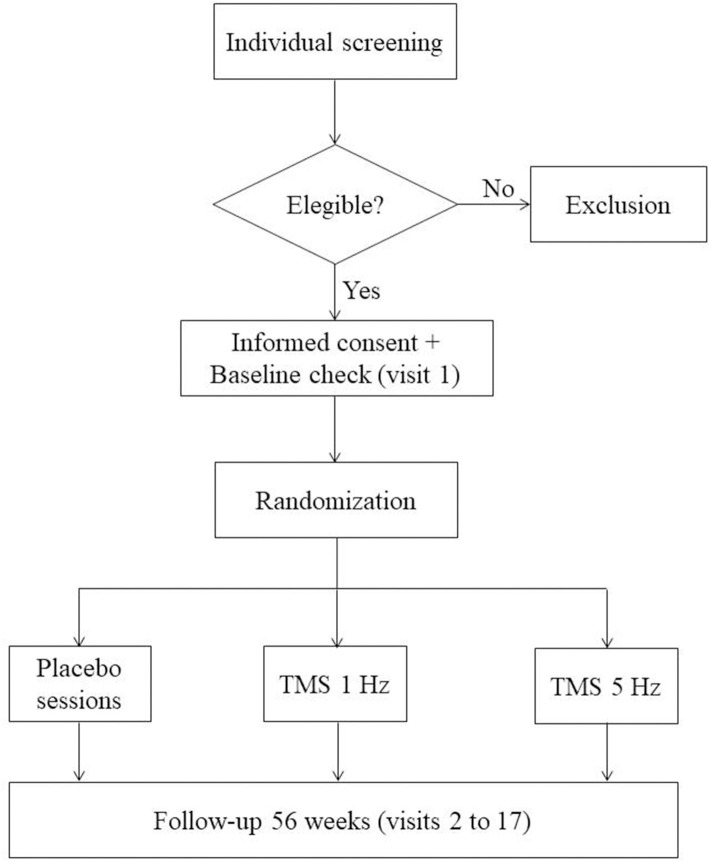
CONSORT flow diagram. Consolidated Standards of Reporting Trials (CONSORT) flow diagram for enrollment and randomization GOAL study.

Phase II clinical trial, unicentric, controlled, randomized, single blind. A Consolidated Standards of Reporting Trials (CONSORT) flow diagram for enrollment and randomization in the GOAL study is showed in Figure 1. The patients included will be assigned under the 1:1:1 randomization formula, constituting three groups for the present study: 30 patients treated with natalizumab + white (placebo) + 30 patients treated with natalizumab + TMS (1 Hz) + 30 patients treated with natalizumab + TMS (5 Hz).

### Eligibility Criteria/Participants

Participants will be recruited from the Reina Sofía University Hospital (Córdoba, Spain). Ninety patients diagnosed with RRMS, who meet all the inclusion criteria and do not present any of the exclusion criteria that are established below and from which clinically evaluable results can be obtained. In Table 1, we present the study's inclusion and exclusion criteria. Patients in the three RRMS groups are treated with natalizumab. It could have been decided to recruit those treated with another pharmacological therapy (such as alemtuzumab, being a more modern monoclonal antibody); however, natalizumab offers us four advantages: (i) Its idiosyncrasy of administration (intravenous) leads to the patient going to the hospital and undergoing a blood analysis, which facilitates obtaining the sample needed in the present study. (ii) It is the drug with which a greater number of patients with RRMS is currently being treated in our hospital, which makes it possible to maximize the possibilities of recruitment. (iii) Regarding the previous point, considering patients under the same treatment (pharmacological) allows us to homogenize the characteristics of the three groups of the sample recruited, which increases the internal and external validity of results. (iv) To the above we can add that natalizumab is the drug with which the research group has the most experience. (v) In our healthcare area, it is the drug that allows us greater recruitment capacity and therefore greater viability to carry out the study (47–50).

**Table 1:** Inclusion and exclusion criteria for the study.

**Inclusion criteria**	**Exclusion criteria**
• Patients diagnosed with RRMS in their inflammatory forms who have completed a 14-dose treatment with natalizumab. • Normal analytical parameters, defined by: Leukocytes>3,000/mcl, Neutrophils>1,500/mcl, Platelets>100,000/mcl, AST/ALT <2.5 IU/L, Creatinine <2.5 mg/dl. • Patients of both sexes aged between 18 and 60 years. • EDSS: between 3.0 and 6.5 points. • Patients who give their informed consent for participation in the clinical trial. • Women of childbearing potential must obtain negative results in a pregnancy test performed at the time of inclusion in the study and commit to using a medically approved method of contraception for the duration of the study.	• Any active or chronic infection, including HIV infection, or hepatitis B or C. • History of neoplasia (basal cell carcinoma of the skin and *in situ* carcinoma in remission are excluded for more than 1 year). • Life expectancy severely limited by other co-morbidities. • Endocrine disease such as diabetes, hyper, or hypothyroidism. • Chronic inflammatory or autoimmune disease such as ulcerative colitis, Crohn's disease, systemic lupus erythematosus and any other form of connective tissue disease or chronic arthropathy. • Chronic obstructive pulmonary disease. • Severe psychiatric illnesses. • Hepatic, or renal, or cardiac dysfunction (including coronary heart disease and heart failure). • Chronic anemia. • Pregnancy or risk of pregnancy (including refusal to use contraception). • Women in breastfeeding period. • Inability to undergo MRI scans. • Inability to grant written informed consent. • Taking lipid-lowering drugs and vitamin supplements. • Treatment with steroids and/or non-steroidal anti-inflammatories, or alcohol intake 40 h before the blood extraction and/or development of the different tests. • Chronic enolism and/or abuse of drugs of abuse (sporadic or chronic). • Metallic implants in the head. • Cardiac pacemaker device.

*RRMS, Relapsing-Remitting Multiple Sclerosis; EDSS, Expanded Disability Status Scale*.

### Identification of the Participants

Each patient will be identified with an alphanumeric code that will be assigned according to his/her correlative order of inclusion (when the patient signs the informed consent). In case of one patient voluntarily withdraws from the study, or in the event that after being included in the trial (upon signing the consent form) the selection criteria are reviewed and it is considered as unfit to continue the study, his/her identification code it cannot be reused for another patient. Therefore, the identification code of each patient will be unique in any way.

Members of research group will only be able to identify the subjects by the code assigned to them, their date of birth and their gender. The principal investigator must keep a confidential record with the patients' names and their assigned identification codes.

### Evaluation of Patients

The main goal is to demonstrate the therapeutic effect of TMS in patients with MS by means of measurement of clinical changes according to the Expanded Disability Status Scale (EDSS). Consequently, the specific objectives are:

- To determine the consequences of the administration of TMS (1 Hz/5 Hz) in patients with MS, paying special attention to its clinical impact according to the MSFC scale (Multiple Sclerosis Functional Composite).
- To assess the effect of the application of TMS (1 Hz/5 Hz) on fatigue in people with MS, according to the FIS scale (Fatigue Impact Scale).
- To observe the effect of the application of TMS (1 Hz/5 Hz) on the degree of depression, according to the Beck scale.
- To study the impact of TMS (1 Hz/5 Hz) on cognitive changes in patients with MS, in relation to the BRB scale (Brief Repeatable Battery of Neuropsychological Test).
- To identify the changes induced by the application of TMS (1 Hz/5 Hz) on neurochemical biomarkers, oxidative damage, acute phase reactants, and in differential expression proteomic profiles, in patients affected by MS.
- To establish the possible associations between the parameters studied and the likely changes that may be observed in them after TMS therapy.

For these purposes, participants will be assessed through an extensive evaluation including blood tests, image analysis, and cognitive functioning, fatigue and depression degree using several scales (Table 2). As it is exposed in the evaluations schedule (in the timeline of every evaluation and variables to examine) (Table 3), the period of follow-up per patient is 56 weeks (around 13 months). Given that the project is designed for 36 months, the idea is that the inclusion of patients is staggered during the first 18 months, at a rate of 5 monthly patients, so that at that time (18 months) the first patients have concluded their period of treatment and clinical assessment and at 24–30 months the field work concerning clinical examinations of patients is concluded. The laboratory studies (determination of routine biochemical magnitudes, laboratory parameters, and proteomics studies) will be carried out in parallel with the recruitment of patients, while the biological samples are obtained. In this way, the work of the entire research team will be continuous from the beginning to the final assessment of the last patient recruited. After that (months 24–30), that is, around 40 months, the statistical study would be carried out, until the conclusion of the proposed study at 36 months.

**Table 2:** Variables to study.

**EVALUATION BATTERY DURING THE PROPOSED TRIAL**
**Clinical parameters**
• Activity degree of the disease: Expanded Disability Status Scale (EDSS). • Comprehensive clinical assessment of the disease: Multiple Sclerosis Functional Composite (MSFC). • Cognitive function: Brief Repeatable Battery of Neuropsychological Test (BRB). • Assessment of fatigue: Fatigue Impact Scale (FIS). • Depression assessment: Beck depression scale.
**Image analysis**
•Radiological study: Nuclear Magnetic Resonance (NMR) with and without contrast.
**Laboratory study**
•Hematimetry and routine biochemistry analysis (glucose, lipid profile, total proteins, albumin, transaminases, CK and LDH). • Biomarkers of oxidative damage: lipoperoxidation products and plasma carbonylated proteins. • Redox state of glutathione (total glutathione, GSH, GSSG and GSH/GSSG ratio). • Levels of neurotrophic factors (BDNF and NGF). • Cytokines: TNF-alpha.

**Table 3:** Schedule of visits and parameters to check.

**Visit number**	**1**	**2**	**3**	**4**	**5**	**6**	**7**	**8**	**9**	**10**	**11**	**12**	**13**	**14**	**15**	**16**	**17**
Week number	(^*^)	−4	0	4	8	12	16	20	24	28	32	36	40	44	48	52	56
Informed consent	X																
Clinical record	X	X	X	X	X	X	X	X	X	X	X	X	X	X	X	X	X
General examination	X		X	X	X	X	X	X	X	X	X	X	X	X	X	X	X
Vital signs (BP, HR, BR)	X		X	X	X	X	X	X	X	X	X	X	X	X	X	X	X
Neurological examination	X								X						X	X	X
Absence outbreaks	X	X	X	X	X	X	X	X	X	X	X	X	X	X	X	X	X
Neuropsychological study	X								X						X	X	X
EDSS scale	X		X						X						X	X	X
MSFC scale	X								X						X	X	X
Routine laboratory tests	X								X						X	X	X
Quality of life scale	X								X						X	X	X
Inclusion criteria	X	X															
Exclusion criteria	X	X															
Adverse effects	X	X	X	X	X	X	X	X	X	X	X	X	X	X	X	X	X
Other previous therapies	X	X	X	X	X	X	X	X	X	X	X	X	X	X	X	X	X
Therapy in trial (TMS/Placebo)			X	X	X	X	X	X	X	X	X	X	X	X	X		
Motor threshold		X															

* *Start time (recruitment moment); BP, Blood Pressure; HR, Heart Rhythm; BR, Breathing Rate; EDSS, Expanded Disability Status Scale; MSFC, Multiple Sclerosis Functional Composite; TMS, Transcranial magnetic stimulation*.

### Sample Size

Lacking precedents in the scientific literature on the use of TMS in MS, we have based our work on three premises to estimate the sample size: (1st) Taking as a reference the dimensions of the groups of patients referred in clinical trials in which TMS has been applied to other neurodegenerative diseases. (2nd) The main objective of the trial is to achieve clinical improvement of the patient. In the absence of outcome variables of a clinical nature, surrogate variables (indirect outcome indicators) could be considered, such as some of the parameters of oxidative stress. In this case, if we take the redox ratio (GSH/GSSG) as a dependent variable, its standard deviation is 0.26 and the minimum expected difference of 10. With an error α = 0.05, an error ß = 0.20 and estimating a 20% loss of follow-up, at least 10 patients are required for each group (x 3 groups = 30 subjects in total). Note: There is a higher prevalence of MS in females, so it is assumed that according to the sampling technique, in the three groups considered there will be a similar gender ratio. It is also assumed that the response to TMS will not be conditioned by the patient's gender, which would work to improve the external validity of the results. (3rd) Being a phase II clinical trial, the initial piloting could justify the establishment of a number of subjects according to the investigator's criteria, according to their experience and the “state of the art” of the technique, allowing for a reduced number of patients.

### Statistical Analyses

The feasibility analysis will be done by intention to treat and will include all patients for whom we have some feasibility data. The qualitative variables will be expressed by absolute and percentage frequencies, while the quantitative variables will be presented through the mean, median, standard deviation, maximum, minimum and number of observations. All *p*-values and confidence intervals will be calculated and evaluated using a 95% confidence level. The main effectiveness variable will be analyzed in the following way: the comparison of means of the values obtained in the quantitative variables studied during the successive visits, will be carried out through the ANOVA test of repeated measures if the data allow to apply a parametric statistics (as follows a normal distribution) and/or by the Friedman test (as a non-parametric equivalent). The evaluation of the qualitative parameters will be carried out through the Chi-square test.

### Possible Loss of Patients: Withdrawal Criteria and Analysis of Anticipated Loss and Abandonments

A withdrawal is defined as the situation in which a subject included in the Clinical Trial ends his/her participation in it before completing the protocol in its entirety, independently of the circumstances that motivate the termination. Patients will interrupt their participation and will be removed from the clinical trial in any of these situations: (i) Presence of a serious adverse event since the patient's recruitment. (ii) Clinical conditions of the patient that prevent his/her continuity. (iii) Other reasons: protocol violation, lack of cooperation, revocation of informed consent, loss of follow-up.

The date and reason why a subject interrupts his/her participation in the Clinical Trial must be recorded in the Data Collection Notebook. The circumstance of the interruption should be notified immediately to the monitor and if this has been a Serious Adverse Event. The patient has the full right to leave the study at any time and any patient can be removed from the study for any reason beneficial to his/her well-being. According to the standards of Good Clinical Practice (GCP), all patients who leave the study before the foreseen time will be recommended the best alternative treatment.

### Contingency Plan

This has been planned from a preventive point of view. Therefore, before starting it was assumed up to 20% loss of patients' follow-up in the calculation of the sample size.

### Interruption of the Clinical Trial

The trial will be interrupted if any of the following circumstances are met: (i) Serious treatment-related toxicity, as the appearance of seizures following the application of TMS. In this case, when it happens to 50% of the patients enrolled in the study. (ii) Lack of adherence to treatment, as the abandonment of more than 60% of the recruited population. (iii) If one patient dies for reasons directly related to TMS.

### Intervention/Experimental Setup

By means of the magnetic stimulator with the coil located in the primary motor cortex we induce a cerebral electric current that is able to obtain a motor potential in the first dorsal interosseous bone (PID) of the left hand. We will measure muscle stimulation by placing conventional surface electrodes connected to a device of evoked potentials. It is a Compound Muscle Action Potential (PAMC) that represents the sum of the action potentials of all the individual muscle fibers underlying the electrodes. For this, the electromyograph is programmed with the following parameters: (A) Sensitivity: 50 uV; (B) Frequency filter: between 2,000 Khz and 1 Hz; (C) Scan speed 10 ms/div; (D) Digitized preamplification signs, and (v) Surface electrodes are placed: active in the eminence of the dorsal interosseous muscle and reference in the dorsal bony prominence of the second finger. The procedure will depend on the group to which the patient has been assigned. In groups 2 and 3, we will place the probe from 8 to 3 centimeters in front of the vertex (Cz) medially and perpendicular to the craniocaudal axis.

The intervention procedure consists of two steps: First step: Obtaining the Motor Evoked Threshold at Rest: Regardless of to the group to which they belong, each patient will have his/her threshold evoked motor calculated at rest, by stimulation of the motor cortex in both hemispheres, evoking electromyographic responses (EMG) in the contralateral muscles, called motor evoked potentials (MEP). The threshold of motor excitability at rest (TM) is defined as the minimum intensity (expressed as the percentage of the maximum output power of the stimulator) capable of producing a reproducible MEP in a resting muscle in 50% of 10 shots. Second step: Administration of the Transcranial Magnetic Stimulation: A Rapid 2 Magstim device (Magstim Co.®, Whitland, Carmarthenshire, Wales) equipped connected to a figure-of-eight coil of 70 mm will be used. This equipment is used to calculate the TM and the percentage of it to which the treatment will have to be applied. The selection of the specific point of stimulation in the Supplementary Motor Area (SMA) will be sufficiently anterior to prevent the propagation of the impulse from triggering the muscular contraction of the shoulders, trunk and lower limbs. The position of the coil will be marked on the scalp to ensure consistent placement of the coil throughout the experiments, the patient will be fitted with a lycra cap on which to indicate and mark also the exact stimulation point, as well as on which to place and hold the coil during the therapeutic session. The coil will be oriented toward the posterior area in order to trigger a postero-anterior current. The TM will be calculated in relation to the evoked potential and according to international standards; based on it, TM is defined as the lowest stimulus intensity that elicited a minimum MEP amplitude of 50 mV in the completely relaxed FDI muscle in at least 5 out of 10 consecutive trials (51, 52). TMS will be applied through a non-ferromagnetic figure-ofeight coil (70-mm outer wing diameter) connected to a Magstim Rapid stimulator (The Magstim Co.®, Wales, UK) which generates biphasic electrical pulses of ~250 ms duration, at 90% of each individual's motor threshold at a frequency of 1 Hz or 5 Hz depending on the group, applying a total of 900 pulses, distributed in three sessions of 300 pulses with a 10 min pause between each session and a total duration of 45 min. Its location will be 3 cm ahead of the midpoint (Cz) on the cranio-caudal axis to simultaneously stimulate the SMA of both hemispheres, bearing in mind that the RRMS is characterized by diffuse lesions in both hemispheres. The treatment will be administered for 5 consecutive days, with 3 weeks of rest, between each stimulation. To complete a treatment period of 14 months (based on previous studies of the group in RRMS patients treated with natalizumab). In the case of the placebo group (patients with RRMS treated with natalizumab and placebo coil) patients will be stimulated with an inactive probe, the perception being indistinguishable. The stimulation with TMS (or administration of placebo) will be carried out every day in the same time slot for 5 consecutive days every 4 weeks, during a period of 14 months.

## Discussion

After carrying out this study presented, it would possibly have a high scientific and social relevance according to it aims to make a positive, relevant and innovative difference to: (i) its contribution to scientific knowledge and advancement in neurosciences field; (ii) its contribution to generate new tools (especially in therapies for neurodegenerative diseases), models or analysis systems that could enable improvement, boosting or creation of scientific research fields; (iii) its contribution to improve the health and well-being of citizens, due to its focus on a high prevalent disabling disease (as multiple sclerosis is); in addition, its incidence is increasing.

In the case of obtaining the expected results, these would involve the design of a new therapeutic strategy for patients with MS. It would be a new treatment that would improve the quality of life and the patient's activity, which would suggest the inclusion of TMS in the therapeutic approach algorithms for MS. Consequently, on the one hand it can lead to the design of a patentable application protocol (when MS is established as a new use), and on the other hand it can contribute to the design of new models of magnetic stimulators in relation to the variants of their physical foundations. Similarly, biomarkers for clinical use (diagnosis and/or prognosis) and therapeutic targets that are identified in the study of molecular profiles related to the clinical variables of these patients would be subsidiary to being patented. Therefore, the possibilities of patentability are high, especially considering the environment of industrial protection and the intellectual protection of the results subsequent to biomedical research. Suffice it to recall that, together with the field of biomaterials, electromechanical devices, and devices (both for diagnostic and therapeutic use) represent the largest source of patents in this research sector. In addition to this (and of special interest in the project proposed here) it would be patentable to establish the use of a device or equipment (already patented) for a new indication. Therefore, although various models of equipment for administration of TMS have already been patented, as of today there are no patents for use in MS. Currently, this technique is approved by the Food and Drug Administration (FDA) and the European Medicines Agency (EMEA) for other indications, as stated in the project application report; however, its application in MS would be the first.

If we rely on the results obtained in other neurodegenerative diseases and in experimental models of MS, we could foresee a clinical improvement of the patients, as well as their molecular profile. In relation to this improvement, such benefits could have an impact at these three levels: (i) Impact at the level of routine clinical care practice: It would involve the design of new therapeutic strategies for, at least, patients with multiple sclerosis. It would be a new treatment that would improve the patient's state and with it the quality of life and the activity of the patient, which would make it a subsidiary to be included in the algorithms of therapeutic approach of the patient with MS; (ii) Impact at the organizational and health resources management level: We are developing a new therapeutic application of a system (TMS) already used in the usual practice of the health system in neurophysiology for the study of potentials and the neurological communication channels. It is also currently approved by both the FDA and the European Drug Agency for the treatment of psychiatric disorders such as depression resistant to conventional treatment; iii) Possible inclusion of the expected results in consensus documents, clinical practice guidelines or care protocols: As previously mentioned, application protocols could be generated, potentially involving their potential inclusion in clinical practice guidelines, as has happened in the administration of TMS in other neurodegenerative and psychiatric processes.

## Ethics and Dissemination

The proposed clinical trial will be conducted in accordance with the protocol following the standard procedures established at the participating hospital. Said trial will be carried out according to the recommendations for Clinical Trials and product evaluation in human research phase, which appear in the Declaration of Helsinki, reviewed in the successive world assemblies (WMA, 2008), and the current Spanish Legislation in the field of Clinical Trials (RD 1090/2015). The ICH-GCP standards (CPMP/ICH/135/95) will be followed. The Clinical Research Ethics Committee (CEIC) of Córdoba has already reviewed and approved the protocol and informed consent in December 2017, as well as the completion of the present clinical trial itself. Before carrying out any of the procedures specified in the protocol, the participating subject must sign and date the informed consent document approved by the CEIC. In order to guarantee the confidentiality of the trial data, the original data will be kept in the hospital and will only be accessed by the researcher and his/her team of collaborators, the trial monitor and the CEIC of Córdoba, which is the body that would protect the present essay. The researcher will allow the audits and inspections of the Spanish or European Health Authorities. The content of the data collection notebooks and the confidentiality of the data of each patient will be respected at all times. Appropriate procedures will be followed to ensure compliance with the provisions of Organic Law 15/99 of December 13 on the Protection of Personal Data. The documents generated during the study, will be protected from uses not allowed by people outside the investigation and, therefore, will be considered strictly confidential and will not be disclosed to other people.

The plan of initial dissemination of the results would include two important and complementary aspects at the same time: (i) Dissemination in biomedical scientific forums: in the first place, the results of this research project will be presented in the National and International Congresses of the Scientific Societies; secondly, the results will be published through peer-reviewed publications. (ii) Social and health dissemination: our research group participates continuously in forums, meetings, conferences and dissemination meetings on neurosciences, neurodegenerative diseases and advances in medicine, and interacts with the associations of patients and relatives of patients with MS.

## Ethics Statement

The Clinical Research Ethics Committee (CEIC) of Córdoba has already reviewed and approved the protocol and informed consent in December 2017, as well as the completion of the present clinical trial itself. Before carrying out any of the procedures specified in the protocol, the participating subject must sign and date the informed consent document approved by the CEIC.

## Author Contributions

EA, JC-V, and IT: conceptualization. EA and MB: methodology. CC: neuropsicological analysis. EA and MB: neurological analysis. JC-V and AG: laboratory analysis. BE and MF: data analysis. EA and JC-V: writing—original draft preparation. IT: supervision. All authors read and approved the final manuscript and take full responsibility for the manuscript content.

## Conflict of Interest

The authors declare that this study protocol was designed in the absence of any commercial or financial relationships that could be construed as a potential conflict of interest.
